# The Association Between Severe Mental Illness and Receipt of Acute Cardiac Care for Myocardial Infarction, and the Impact of the COVID-19 Pandemic

**DOI:** 10.1192/bjo.2024.88

**Published:** 2024-08-01

**Authors:** Kelly Fleetwood, Stewart Mercer, Sandosh Padmanabhan, Daniel Smith, Caroline Jackson

**Affiliations:** 1 University of Edinburgh, Edinburgh, United Kingdom; 2 University of Glasgow, Glasgow, United Kingdom

## Abstract

**Aims:**

To compare receipt of acute cardiac care in people with versus without severe mental illness (SMI) and investigate the impact of the COVID-19 pandemic on any differences in care. We hypothesised that, compared with those without SMI, patients with an SMI are less likely to receive guideline recommended acute cardiac care and that disparities worsened as a result of the pandemic.

**Methods:**

We conducted a cohort study using data from the CVD-COVID-UK resource, which links electronic health data from multiple sources. Our cohort included 95,125 adults with a non-ST-elevation MI (NSTEMI) recorded in the Myocardial Infarction National Audit Programme (MINAP) dataset between 1 November 2019 and 31 March 2022. We defined SMI as schizophrenia, schizoaffective disorders or bipolar disorder (BD), ascertained through recorded diagnosis in primary care or hospital admission records. We examined receipt of cardiac care standards for NSTEMI, including: admission to a cardiac ward; angiogram eligibility; receipt of angiogram (in those eligible); angiogram within 72 hours; secondary prevention medication prescribing at discharge, and arrangement of post-discharge cardiac rehabilitation. We used logistic regression to obtain odds ratios (ORs) for the association between SMI and receipt of each care indicator, adjusting for age, sex and time period. We tested for an interaction between SMI and time period in order to determine if any disparities had changed since the start of the COVID-19 pandemic.

**Results:**

Within our cohort, 620 patients (0.6%) had schizophrenia and 575 (0.6%) had BD. Compared with people without SMI and after adjusting for age, sex and period, patients with an SMI were less likely to receive each of the cardiac care standards. For example, compared with those without SMI, those with SMI were less likely to: be admitted to a cardiac ward (schizophrenia: OR 0.72, 95% CI 0.61–0.85; BD: 0.74, 95% CI 0.63–0.88); be eligible for an angiogram (schizophrenia: 0.37, 95% CI 0.29–0.47; BD: 0.52, 95% CI 0.40–0.68); receive an angiogram (schizophrenia: 0.22, 95% CI 0.18–0.28; BD: 0.51, 95% CI 0.39–0.66); and receive an angiogram within 72 hours (schizophrenia: 0.71, 95% CI 0.56–0.90); BD: 0.80, 95% CI 0.64–1.00). We generally found no evidence that disparities had changed since the start of the COVID-19 pandemic.

**Conclusion:**

We identified marked SMI disparities in receipt of acute cardiac care among people treated in hospital for a NSTEMI. Further research should seek to identify reasons for, and inform interventions to, address these disparities.

